# Oleic acid released by sensory neurons inhibits TRPV1-mediated thermal hypersensitivity via GPR40

**DOI:** 10.1016/j.isci.2024.110552

**Published:** 2024-07-20

**Authors:** Maksim Sendetski, Saskia Wedel, Kenta Furutani, Lisa Hahnefeld, Carlo Angioni, Jan Heering, Béla Zimmer, Sandra Pierre, Alexandra-Maria Banica, Klaus Scholich, Sorin Tunaru, Gerd Geisslinger, Ru-Rong Ji, Marco Sisignano

**Affiliations:** 1 Goethe University Frankfurt, University Hospital, Institute of Clinical Pharmacology, Theodor-Stern-Kai 7, 60590 Frankfurt Am Main, Germany; 2 Department of Anesthesiology, Center for Translational Pain Medicine, Duke University Medical Center, Durham, NC, USA; 3 Fraunhofer Institute for Translational Medicine and Pharmacology ITMP, Theodor Stern-Kai 7, 60596 Frankfurt Am Main, Germany; 4 Cell Signalling Research Group, Institute of Biochemistry of the Romanian Academy, Splaiul Independentei 296, 060031 Bucharest, Romania; 5 Departments of Cell Biology and Neurobiology, Duke University Medical Center, Durham, NC, USA; 6 Fraunhofer Cluster of Excellence for Immune-Mediated Diseases (CIMD), Theodor Stern-Kai 7, 60596 Frankfurt Am Main, Germany

**Keywords:** Biological sciences, Neuroscience, Molecular neuroscience, Sensory neuroscience

## Abstract

Noxious stimuli activate nociceptive sensory neurons, causing action potential firing and the release of diverse signaling molecules. Several peptides have already been identified to be released by sensory neurons and shown to modulate inflammatory responses and inflammatory pain. However, it is still unclear whether lipid mediators can be released upon sensory neuron activation to modulate intercellular communication. Here, we analyzed the lipid secretome of capsaicin-stimulated nociceptive neurons with LC-HRMS, revealing that oleic acid is strongly released from sensory neurons by capsaicin. We further demonstrated that oleic acid inhibits capsaicin-induced calcium transients in sensory neurons and reverses bradykinin-induced TRPV1 sensitization by a calcineurin (CaN) and GPR40 (FFAR1) dependent pathway. Additionally, oleic acid alleviated zymosan-mediated thermal hypersensitivity via the GPR40, suggesting that the capsaicin-mediated oleic acid release from sensory neurons acts as a protective and feedback mechanism, preventing sensory neurons from nociceptive overstimulation via the GPR40/CaN/TRPV1-axis.

## Highlights

• Nociceptive stimulation of DRG neurons with capsaicin induces oleic acid release
• Oleic acid inhibits TRPV1 activity through desensitization
• GPR40 (FFAR1) and calcineurin are involved in oleic acid’s regulation of induced TRPV1
• GPR40 is required for oleic acid-mediated reduction of thermal hypersensitivity *in vivo*

## Graphical abstract

**Figure:**
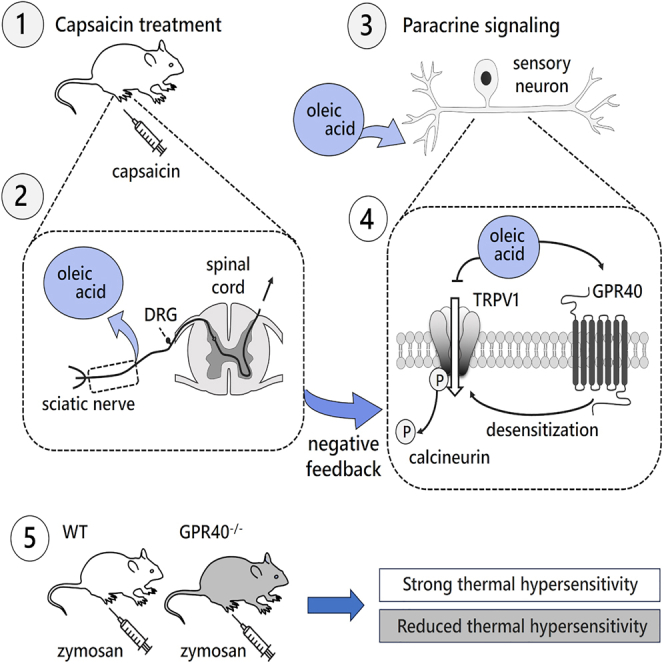

## Introduction

Pain is experienced when a mechanical, thermal or chemical external stimulus exceeds a certain threshold and causes an activation of peripheral sensory neurons.^1^ To “sense” these external stimuli, sensory neurons express a wide range of membrane-bound transducer channels that can detect external changes and respond to noxious stimuli by increasing their open probability to cause a rapid calcium influx into the neurons. This can lead to depolarization and the generation of action potentials.^2^

One of the most important transducer channels in the context of pain belong to the group of transient receptor potential (TRP) channels. Within this group, the TRPV1 (vanilloid) channel, also called the capsaicin receptor, is the best-characterized member.^3^^,^^4^ Although the atomic structure of these channels has been determined and their role in the pathophysiological processes, which can lead to chronic pain, identified, the development of small molecule TRP channel inhibitors has unfortunately been unsuccessful until now, since TRPV1 is additionally involved in the physiological body temperature regulation.^5^ However, since millions of people suffer from chronic pain worldwide and get treated with inadequate therapeutic options,^6^ there is an emerging need for the development of novel analgesics and new approaches.

Sensory neurons respond to painful stimuli not only by depolarization and action potential firing, but also by releasing various signaling mediators. Nevertheless, only few of these mediators have been identified and their roles in pain processing remain largely unknown. First evidence for these signaling mediators was the identification of peptides that are released from sensory neurons to warn immune cells and to initiate inflammation.^7^ Moreover, sensory neurons can suppress immune cell responses by releasing the neuropeptide calcitonin-gene-related peptide (CGRP) in the context of bacterial lung infections.^8^ These observations indicate that peripheral sensory neurons communicate with their cellular environment to a much greater extent than previously assumed by releasing various signaling molecules in the context of pain and inflammation. However, there is no information available on the release of lipids from sensory neurons. Lipids can act as paracrine signaling mediators and are known to play an essential role in onset, maintenance, and resolution of pain.^9^^,^^10^^,^^11^

Here, we tested the hypothesis that lipids are released from sensory neurons upon nociceptive stimulation and can alter the activity of cells in their proximity. Using a high-resolution mass spectrometry-based approach (LC-HRMS), we observed that a transient stimulation of sensory neurons by capsaicin is sufficient to release several lipids. The strongest elevation was observed for oleic acid in extracellular concentrations. Oleic acid can reduce capsaicin-induced responses in sensory neurons and can decrease the frequency of capsaicin-induced spinal excitatory postsynaptic currents. Moreover, oleic acid can reduce bradykinin-induced sensitization of TRPV1 in sensory neurons, via the free fatty acid 1 receptor (FFAR1/GPR40). Finally, we show that oleic acid can reduce thermal hypersensitivity induced by zymosan in wild type but not in GPR40 deficient mice *in vivo*. These results show that oleic acid is an endogenous lipid mediator that is released upon stimulation of sensory neurons and can inhibit TRPV1 responses in a GPR40-dependent manner. In this context, oleic acid seems to act as an endogenous neuron-to-neuron-negative feedback regulator that can be released transiently to protect sensory neurons from overstimulation.

## Results

### Oleic acid is released from sensory neurons upon nociceptive stimulation

**Figure 1:**
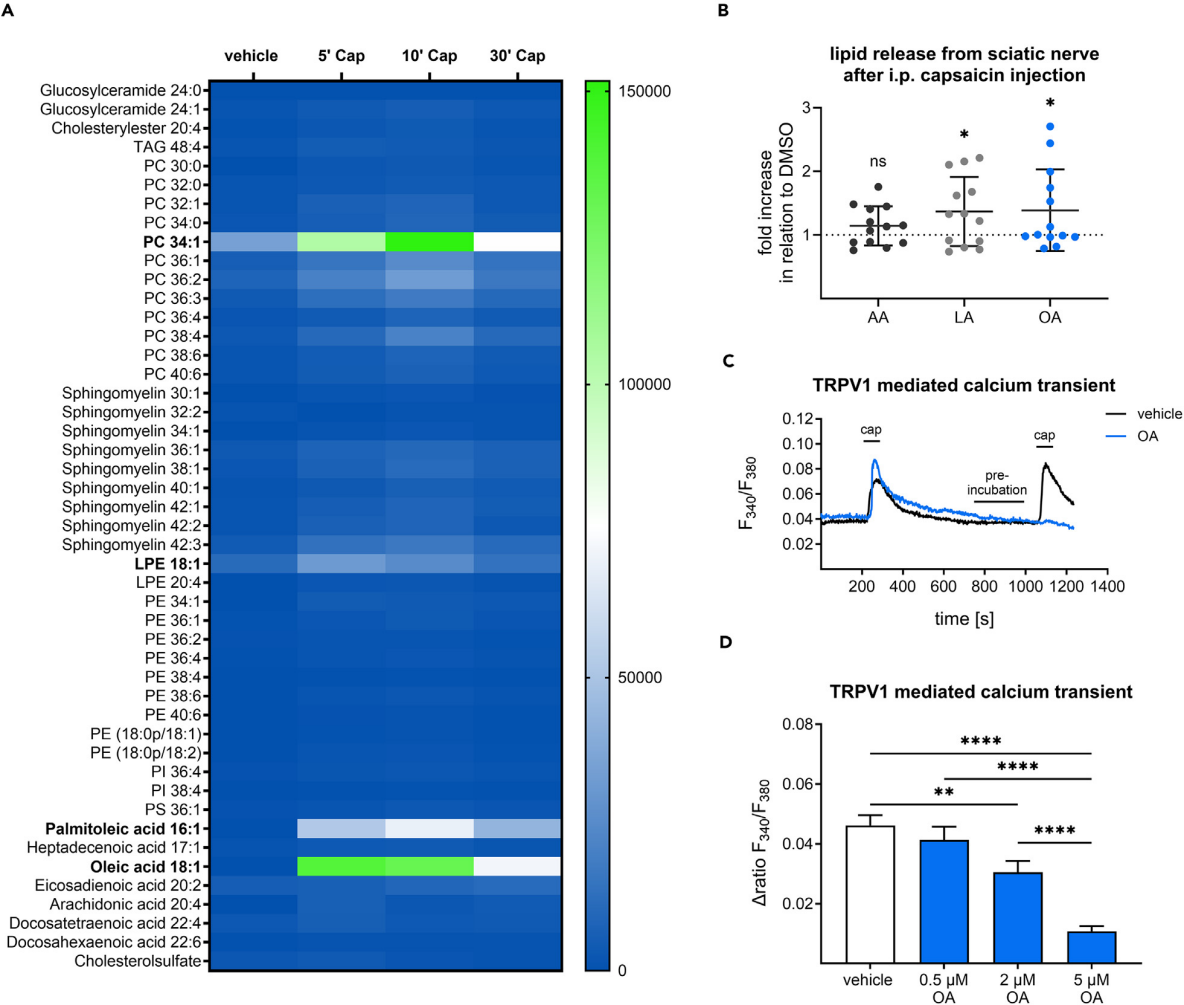
Capsaicin induces TRPV1 desensitizing oleic acid release from DRG neurons and sciatic nerve samples (A) Heatmap depicting the timeline of secreted lipids, as chromatographic peak area values normalized to the respective cell count, in the supernatant of capsaicin treated primary sensory neuron cell cultures. (B) Fatty acid release from sciatic nerves 5 min after intraplantar capsaicin (300 μM). or vehicle (DMSO 1% (v/v)) injection and subsequent tissue dissection. The data represent the mean from 13 sciatic nerves ±SEM. ∗*p* < 0.05 one sample t test. (C) Representative traces of oleic acid-induced reduction of TRPV1-mediated calcium transients in primary sensory neurons using capsaicin (100 nM, 20 s). (D) Quantification of calcium transients after preincubation with oleic acid (5 μM, 2 min). The data represent the mean from 32 to 46 sensory neurons ±SEM. ∗∗*p* < 0.01, ∗∗∗*p* < 0.001, ∗∗∗∗*p* < 0.0001 one-way ANOVA with Tukey’s multiple comparisons test. AA, arachidonic acid; cap, capsaicin; LA, linoleic acid; OA,S oleic acid.

We hypothesized that lipids can be directly released from sensory neurons upon nociceptive stimulation. For this purpose, we used cultured dorsal root ganglion (DRG) neurons from mice and stimulated them with 500 nM capsaicin *in vitro*. An LC-HRMS analysis of the supernatant showed the release of a variety of lipids, including phosphatidylcholine (PC) 34:1, lysophosphatidyl-ethanolamine (LPE) 18:1 and palmitoleic acid. However, the strongest release was observed for oleic acid 5 and 10 min after capsaicin stimulation (Figure 1A). In addition, we investigated the concentration of fatty acids *ex vivo* by (1) injecting capsaicin into the hind paw of mice, (2) dissecting the sciatic nerves 5 min afterward, and (3) measuring lipid concentrations via LC-MS/MS. Oleic acid (*p* = 0.0487) and linoleic acid (*p* = 0.0308) concentrations were increased in sciatic nerve tissue after capsaicin injection compared to the vehicle control (Figure 1B). The levels of arachidonic acid were not significantly altered (*p* = 0.1168, Figure 1B). Moreover, calcium imaging experiments indicated that oleic acid inhibited concentration dependent (2 μM *p* = 0.0074, 5 μM *p* < 0.0001) the capsaicin-induced calcium transients in DRG neurons (Figures 1C and 1D). This calcium transient reduction was not observed for the other lipids (palmitoleic acid, PC 34:1 and LPE 18:1), which were detected in the LC-HRMS screen (*p* = 0.5295, *p* = 0.5520, *p* = 0.2920, Figure S1).

### Oleic acid suppresses the capsaicin-induced potentiation of excitatory synaptic transmission in spinal dorsal horn neurons

**Figure 2:**
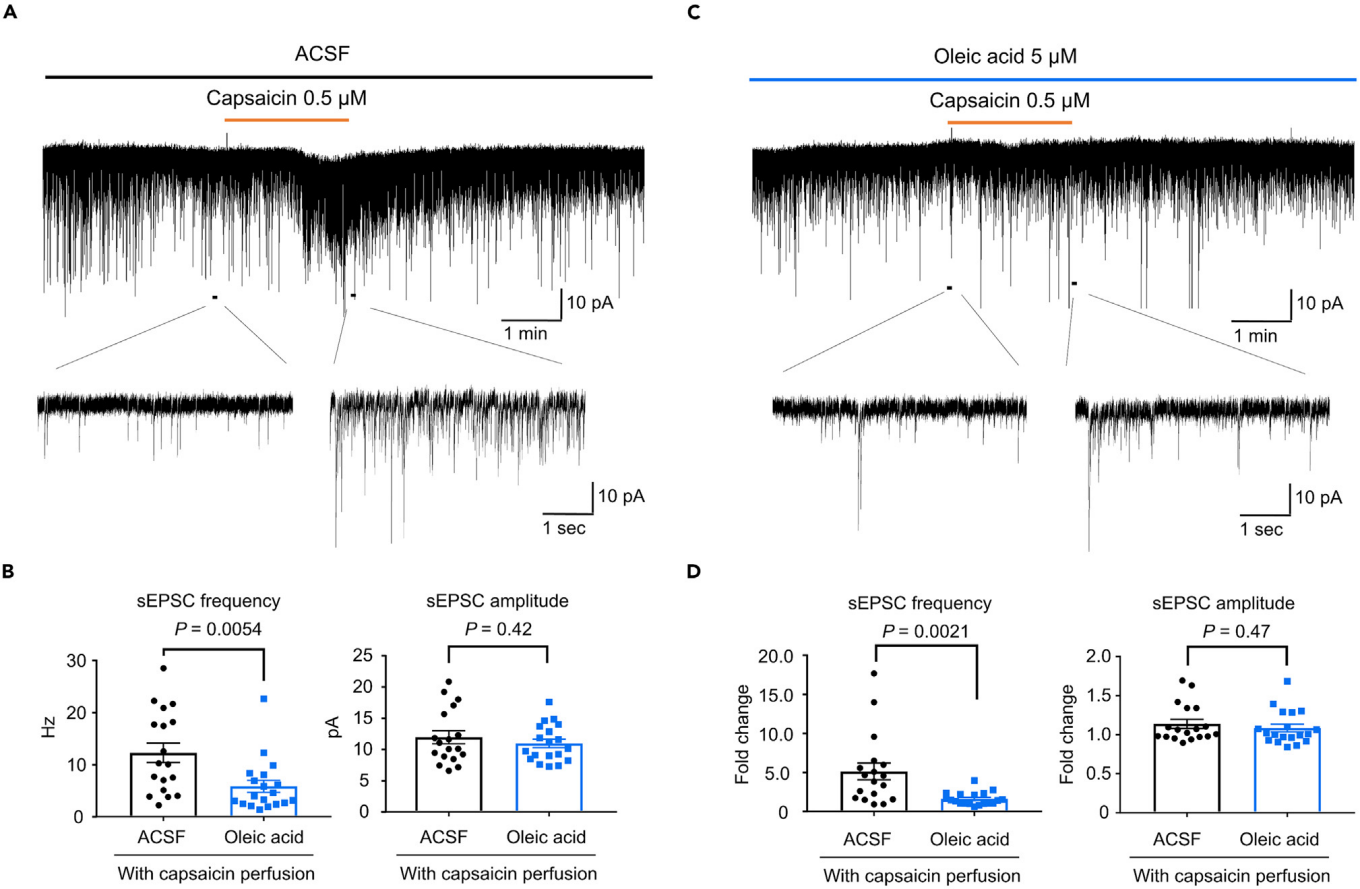
Oleic acid reduces TRPV1-mediated sEPSC frequency in spinal cord slices (A and C) Representative traces of sEPSCs before and after the perfusion of capsaicin (0.5 μM, 2 min), with and without the perfusion of oleic acid (5 μM, during the entire recording). (B) The effects of oleic acid and ACSF on the capsaicin-induced sEPSC frequency (left) and amplitude (right). (D) Influence of oleic acid on capsaicin-induced fold changes on the sEPSC frequency (left) amplitude (right). The data represent the mean of 18–19 neurons from 3 mice ±SEM. ∗*p* < 0.05 unpaired t test. ACSF, artificial cerebrospinal fluid; sEPSC, spontaneous excitatory postsynaptic current.

We employed an *ex vivo* patch-clamp recording approach to study the role of oleic acid in regulating synaptic transmission in spinal cord slices. As previously reported,^12^ bath application of capsaicin (0.5 μM, 2 min) dramatically increased the frequencies of spontaneous excitatory postsynaptic currents (sEPSCs) in outer lamina II (IIo) neurons (Figure 2A). Strikingly, the capsaicin-induced sEPSCs frequency increase was significantly suppressed by co-application of capsaicin with 5 μM oleic acid (oleic acid: 5.8 ± 1.1 Hz; ACSF 12.2 ± 1.8 Hz, *p* = 0.0054, Figures 2C and 2D). We also analyzed the fold change of sEPSC frequency by the treatment in lamina IIo neurons and observed a 5-fold increase by capsaicin in these neurons (Figure 1B). The capsaicin-induced fold increase in sEPSC frequency was also significantly lower in the oleic acid-treated group than the ACSF group (1.62 ± 0.19 vs. 5.14 ± 1.07-fold increase from baseline, *p* = 0.0021, Figures 2B–2D). However, capsaicin had very moderate effects on sEPSC amplitudes (Figure 2B), and furthermore oleic acid (5 μM) had no significant effects on the absolute amplitude of sEPSCs and relative amplitudes (fold change) of sEPSCs after the perfusion of capsaicin (Figure 2D). Additionally, we examined the effects of oleic acid (5 μM) on basal synaptic transmission in the absence of noxious stimulation (capsaicin) and did not observe significant effects of oleic acid (5 μM) on the frequencies (*p* = 0.80) and amplitudes (*p* = 0.092) of sEPSCs (Figure S2).

### Calcineurin and GPR40 mediate the desensitizing effect of oleic acid on TRPV1

**Figure 3:**
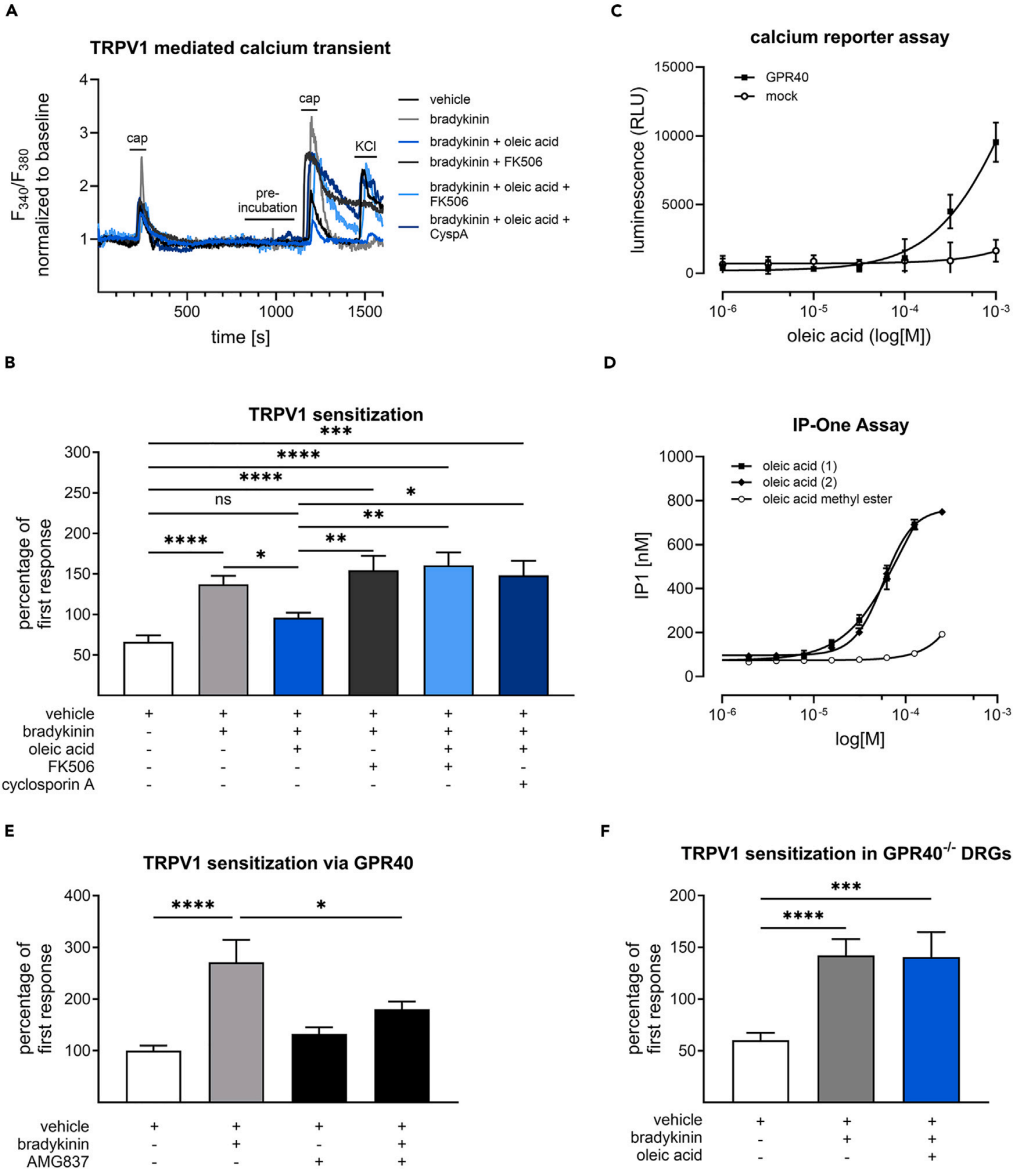
Oleic acid prevents sensitization of the TRPV1 channel via GPR40 in primary sensory neurons (A) Representative traces of TRPV1 mediated calcium transients in sensory neurons, which were pre-incubated with bradykinin (100 nM), oleic acid (1 μM), FK506 (100 nM) and cyclosporin A (100 nM) for 2 min for each condition and stimulated twice with capsaicin (100 nM, 20s). (B) Quantification of TRPV1-mediated calcium transients. The data represent the mean ± SEM of 28–117 primary sensory neurons. ∗*p* < 0.05, ∗∗*p* < 0.01, ∗∗∗*p* < 0.001, ∗∗∗∗*p* < 0.0001 one-way ANOVA with Tukey’s multiple comparisons test. (C) Effect of increasing concentrations of oleic acid on [Ca^2+^]_i_ in CHO cells transiently transfected with cDNAs encoding human GPR40 together with a Ca^2+^-sensitive bioluminescent fusion protein (G5A). The data represent the mean ± SEM of *n* ≥ 3 independent transfections and measurements. (D) Increasing GPR40 activation upon increasing oleic acid concentrations measured by an inositol monophosphate (IP1)-accumulation assay. Data represent the mean ± SEM of *n* = 3 independent transfections and measurements. (E) Effect of the GPR40 agonist AMG837 on bradykinin-induced TRPV1 sensitization. Shown is the mean ± SEM of 22–39 sensory neurons. ∗*p* < 0.05, ∗∗∗∗*p* < 0.0001 one-way ANOVA with Tukey′s multiple comparisons test. (F) Influence of oleic acid on bradykinin-induced TRPV1 sensitization in GPR40 deficient mice depicted as mean ± SEM of 29–85 sensory neurons. ∗∗∗*p* < 0.001, ∗∗∗∗*p* < 0.0001 one-way ANOVA with Tukey’s multiple comparisons test. cap, capsaicin.

**Figure 4:**
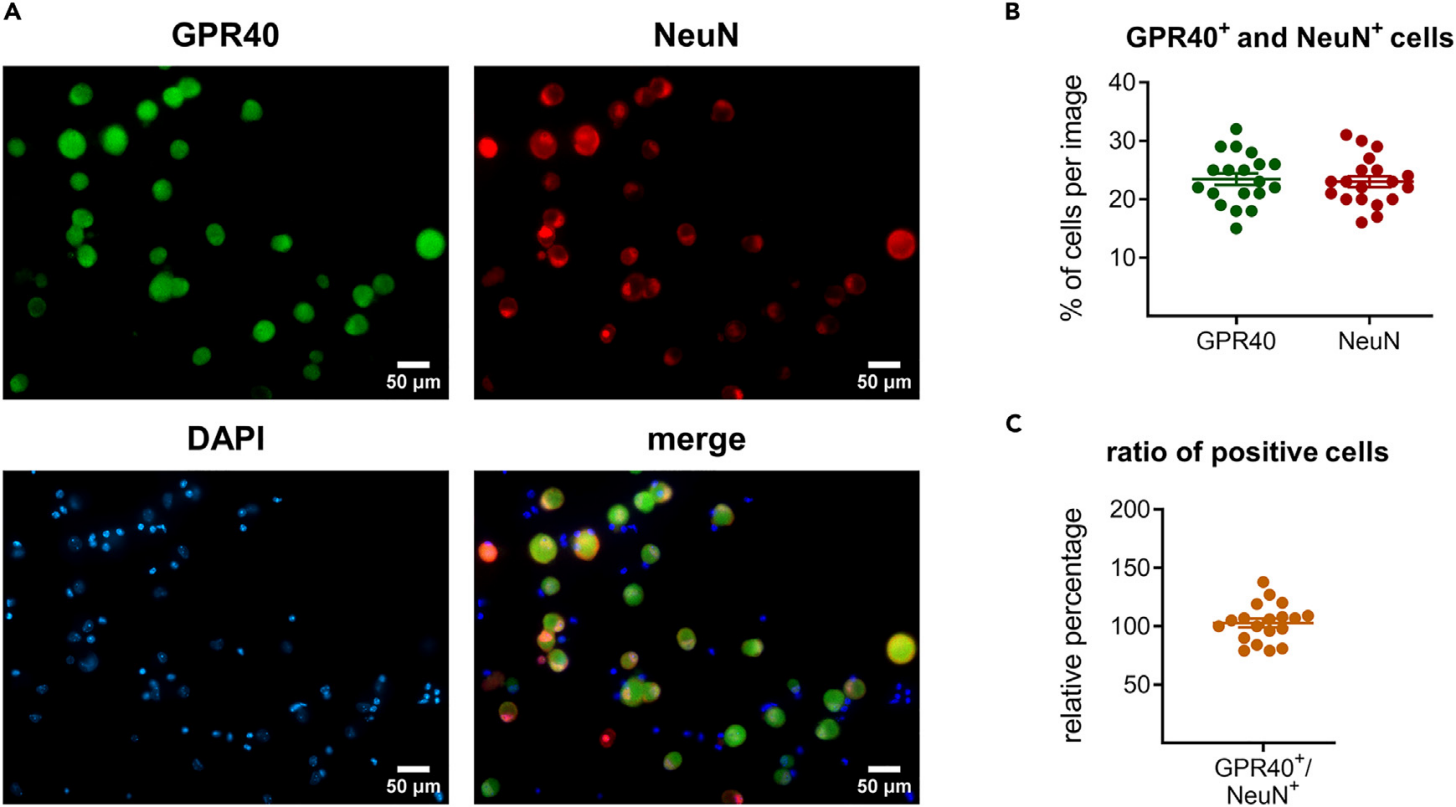
DRG neurons express GPR40 (A) Immunohistochemistry staining of DRG neurons *in vitro* with GPR40, NeuN, and DAPI. (B) Quantification of GPR40 and NeuN positive cells. Data shown as the mean ± SEM of DRG cultures 3 animals. (C) Ratio of GPR40^+^ and NeuN^+^ cells. The data represent the mean ± SEM of DRG cultures 3 animals.

We next hypothesized that oleic acid can affect TRPV1 sensitization that commonly occurs under pathophysiological conditions.^13^ We therefore induced a sensitized state of the TRPV1 channel in murine DRG neurons with bradykinin, which activates the kinin receptors in sensory neurons and causes an increased TRPV1 activity via protein kinase C (PKC).^14^^,^^15^^,^^16^ Using bradykinin, a sensitization of the TRPV1 channel and therefore, an increased capsaicin-induced calcium transient was induced (*p* < 0.0001). This effect was reversed by perfusing the neurons additionally with 1 μM oleic acid (*p* = 0.0037). Since the TRPV1 channel’s sensitization is strongly regulated by its phosphorylation state, we aimed to investigate whether this effect is mediated by calcineurin, which is the most relevant phosphatase in sensory neurons, has previously been identified as the central mediator of TRPV1 activity shifts and desensitization in sensory neurons and can cause antihyperalgesia *in vivo*.^17^^,^^18^^,^^19^ Based on this, we observed that the inhibition of calcineurin (CaN) by two different calcineurin inhibitors, cyclosporine (*p* = 0.0153) and FK506 (*p* = 0.0002), blocked the oleic acid-mediated reversal of the bradykinin-induced sensitization (Figures 3A and 3B). Interestingly, oleic acid has previously been reported to activate the free fatty acid receptors FFAR1 (GPR40). In accordance to this, we verified that oleic acid can activate GPR40 using two different heterologous expression systems of GPR40. The first one uses a calcium-sensitive genetic probe consisting of a fusion protein between aequorin and GFP (Figure 3C) and the second one uses IP-One accumulation as GPR40 activation readout (Figure 3D). In both assay systems, we observed that oleic acid is able to effectively activate GPR40 in living cells. Interestingly, introducing a methylation at the carboxylic group of oleic acid completely disabled the ability of oleic acid to activate GPR40 (Figure 3D), suggesting that the carboxylate group is essential for GPR40 activation. Furthermore, calcium imaging experiments revealed that the perfusion of murine DRG neurons with bradykinin together with the GPR40 agonist AMG837 (1 μM) reversed the bradykinin-induced sensitization of TRPV1 (*p* = 0.0256, Figure 3E) in a similar fashion as we have seen with oleic acid (Figures 3A and 3B). Moreover, we observed that bradykinin can reverse a desensitized to a sensitized state (*p* < 0.0001) in DRG neurons from GPR40 deficient mice, which was not affected by oleic acid (*p* = 09974, Figure 3F). In line with this finding, bradykinin-induced TRPV1 sensitization cannot be reduced when oleic acid is applied together with the GPR40 antagonist DC260126 (Figure S3). To investigate the expression and abundance of GPR40 in sensory neurons, we performed immunostaining of DRG neurons (Figure 4), after they were measured in calcium imaging experiments and responded to oleic acid.

### GPR40 is required for the oleic acid-mediated alleviation of zymosan-induced thermal hypersensitivity

**Figure 5:**
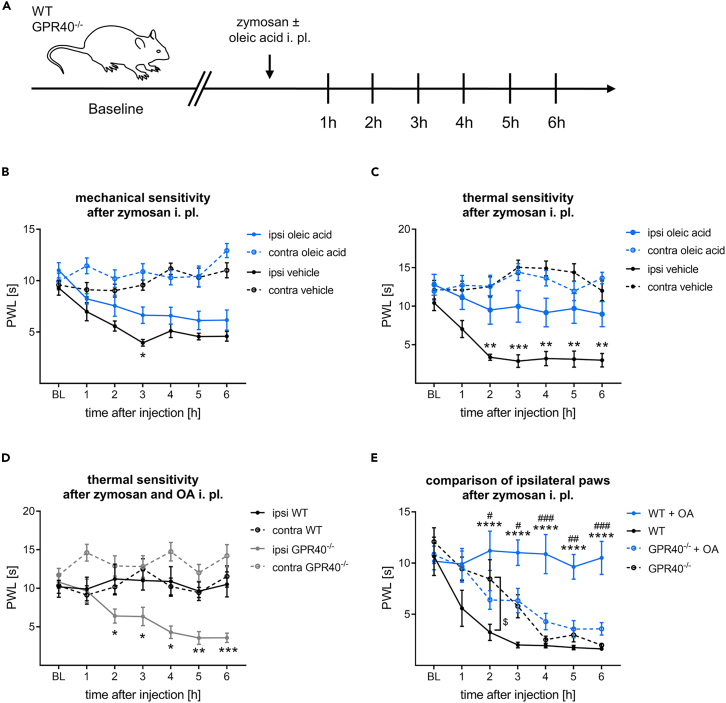
Intraplantar injection of oleic acid protects WT but not GPR40 deficient mice from zymosan-induced thermal hypersensitivity (A) Schematic illustration of behavioral testing and injection protocol. (B) Mechanical pain threshold of mice measured as latency time to mechanical stimuli after intraplantar injection of 3 mg/mL zymosan together with either 50 μM oleic acid or the respective vehicle. The data represent the mean ± SEM of 8 mice per group. (C) Thermal sensitivity of mice after intraplantar injection of 3 mg/mL zymosan together with either 50 μM oleic acid or the respective vehicle. Data are shown as mean ± SEM of 11 mice per group. (D) Comparison of thermal hypersensitivity from wild-type mice with GPR40 deficient mice after intraplantar injection of zymosan and oleic acid. Shown is the mean ± SEM of 8 mice per group. (B, C, and D) ∗*p* < 0.05, ∗∗*p* < 0.01, ∗∗∗*p* < 0.001 two-way repeated measures ANOVA followed by Tukey’s multiple comparisons test. (E) Comparison of thermal pain thresholds from ipsilateral paws of wild-type and GPR40 deficient mice after intraplantar injection of zymosan with or without additional treatment with oleic acid. The data represent the mean ± SEM of 6–8 mice per group ±SEM. WT vs. WT + OA: ∗∗∗∗*p* < 0.001; WT + OA vs. GPR40^−/−^ + OA: #*p* < 0.05, ##*p* < 0.01, ###*p* < 0.001; WT vs. GPR40^−/−^: $*p* < 0.05 two-way repeated measures ANOVA followed Tukey’s multiple comparisons test. contra, contralateral; i.pl., intraplantar; ipsi, ipsilateral; OA, oleic acid; WT, wild-type.

We next assessed whether oleic acid has an effect *in vivo* and whether it can alleviate hypersensitivity in mice. We therefore performed a zymosan-induced acute inflammatory pain model that causes a transient inflammation and inflammatory pain after intraplantar injection.^20^ In addition to zymosan, the mice were additionally injected with oleic acid (50 μM) or vehicle. Afterward, the mechanical or thermal pain threshold was measured hourly for up to 6 h (Figure 5A). Mechanical hypersensitivity was mostly unaffected by oleic acid. Only after 3 h, oleic acid slightly reduced mechanical hypersensitivity (*p* = 0.0471, Figure 5B). In contrast, thermal hypersensitivity was strongly ameliorated in oleic acid treated mice over the entire time span (2 h *p* = 0.0042, 3 h *p* = 0.006, 4 h *p* = 0.0059, 5 h *p* = 0.0018, 6 h *p* = 0.0058, Figure 5C). Next, we aimed to investigate whether oleic acid protects mice from thermal hypersensitivity when GPR40 is not present. Hence, we performed the same experiment with GPR40 deficient and wild-type mice. This revealed that GPR40 deficient mice exhibited strong hypersensitivity compared to wild-type mice, even though they were treated with oleic acid (2 h *p* = 0.0343, 3 h *p* = 0.0415,4 h *p* = 0.0013, 5 h *p* = 0.0037, 6 h *p* = 0.0006, Figure 5D), which indicates that GPR40 is required for the oleic acid-induced reduction of thermal hypersensitivity.

Finally, we investigated differences between GPR40 deficient and wild-type mice, comparing the ipsilateral paw withdrawal latencies of zymosan-induced inflammatory pain with and without oleic acid. As shown in Figure 5E, we observed that neither untreated GPR40 deficient mice nor oleic acid-treated GPR40 deficient mice are protected from zymosan-induced thermal hypersensitivity. Comparing the groups with only zymosan injection, GPR40 deficient mice seem to exhibit slightly less hypersensitivity than wild-type mice (*p* = 0.0297). However, at all other time points these two groups do not differ significantly (1 h *p* = 0.1710, 3 h *p* = 0.1836, 4 h *p* = 0.9894, 5 h *p* = 0.9121, 6 h *p* = 0.9973). In conclusion, oleic acid protected wild-type animals from zymosan-induced thermal hypersensitivity (1 h *p* = 0.0688, 2 h *p* < 0.0001, 3 h *p* < 0.0001, 4 h *p* < 0.0001, 5 h *p* < 0.0001, 6 h *p* < 0.0001) but not GPR40 deficient mice (1 h *p* = 0.8834, 2 h *p* = 0.9993, 3 h *p* = 0.9890, 4 h *p* = 0.7424, 5 h *p* = 0.9882, 6 h *p* = 0.7996).

## Discussion

Previous findings demonstrated that the activity of sensory neurons can trigger the release of signaling mediators to modulate inflammatory processes in the context of bacterial infections.^7^^,^^8^ While these effects are mainly mediated by peptides, such as CGRP, it is known that lipid mediators can also be released by various cell types transiently to act as paracrine signaling molecules and to modulate intracellular pathways of target cells and trigger cellular responses.^21^

In the context of pain, this has been shown for prostaglandins and their release from resident or infiltrating immune cells causing sensitization of neuronal ion channels and neuronal activity in general.^22^ However, it hasn’t been explored whether neurons themselves release lipids in response to nociceptive stimuli. This study aimed to analyze the lipid secretome within the first 10 min after capsaicin-mediated TRPV1 stimulation in cultured DRG neurons and 5 min after capsaicin injection in sciatic nerve tissue. Surprisingly, arachidonic acid and its metabolites, previously associated with persistent pain,^9^ were not elevated. Instead, oleic acid was prominently released from both cultured neurons and sciatic nerve tissue.

There is still the possibility that oleic acid is released from glial cells which are also present in the culture. However, there are three reasons why this is highly unlikely. First, we use neuron-enriched cultures which predominantly consist of sensory neurons and glial cells only make up a small part of it. Second, the oleic acid release happens transiently after capsaicin stimulation, indicating a fast TRPV1-dependent response which cannot be triggered in glial cells but only in TRPV1-positive neurons. Third, we also see the release of oleic acid from sciatic nerves after peripheral capsaicin injection in an *ex vivo* approach, indicating that neuronal and axonal activity of nociceptors is required for this release.

The mechanism triggering oleic acid release remains unclear. It can be speculated that TRPV1 activation by capsaicin induces a calcium influx into sensory neurons, enhancing the activity of calcium-dependent phospholipases (PLAs) that release fatty acid residues from membrane lipids. These phospholipases are known to exhibit substrate specificity and each isoform seems to have a preference to bind ester, alkyl ether, or vinyl ether phospholipids.^23^ The specific PLA isoform, responsible for cleaving and releasing oleic acid, is unknown. Currently, the group V phospholipase A2 is a suggested candidate because it can release oleic acid and linoleic *in vitro*^24^^,^^25^ and it is expressed in murine DRGs and spinal cord.^26^ However, its activity changes in response to transient stimulations are yet to be investigated.

In addition, it has previously been described that oleic acid can inhibit TRPV1 probably by direct binding in the vanilloid-binding pocket (VBP) of the TRPV1 channel, thereby stabilizing its closed state and ultimately inhibiting the activity of the channel.^27^ We can confirm that oleic acid has an inhibitory effect on capsaicin-induced currents in the spinal cord. However, we describe for the first time that oleic acid is released from sensory neurons upon capsaicin stimulation to act as an endogenous signaling mediator and to reduce the activity of TRPV1 in sensory neurons. According to our *in vivo* data, GPR40 is required for this process but we cannot rule out that the oleic acid effect is in part caused by a direct inhibition TRPV1 at the concentrations measured. It is conceivable that oleic acid has both effects depending on its concentration and that it acts as an early and transient warning system to reduce TRPV1 activity in peripheral sensory neurons and to protect neurons from overstimulation. In this regard, oleic acid seems to be unique, because most other mono- and polyunsaturated fatty acids, such as eicosapentaenoic acid (EPA) and docosahexaenoic acid (DHA) seem to have rather activator effects on TRPV1.^28^ The activating effects of polyunsaturated fatty acids have also been described for the TRPA1 (transient receptor potential ankyrin 1) channel which is also expressed in sensory neurons.^29^ TRPA1 can be activated by fatty acids with at least three double bonds, and twenty carbon atoms, which applies for EPA but not for oleic acid. Indeed, as reported in the same publication, oleic acid does not influence TRPA1 activity.^30^ It is also possible that oleic acid triggers a dual effect, at the same time blocking TRPV1 and activating FFAR1, thus leading to a stronger TRPV1 blockading effect in GPR40/TRPV1-double positive neurons.

Recently, it was described that pharmacological inhibition or deficiency of GPR40 can exacerbate incisional pain *in vivo*.^31^ However, the authors mainly connect the GPR40 effects with descending inhibition of pain and focus on fatty acid levels in different brain regions. It is conceivable that oleic acid/GPR40 signaling along these pathways might be associated with more long-term and chronic pain states and neuroinflammation, which needs to be addressed by further studies.

Interestingly, our behavioral results show reduced thermal sensitivity to zymosan in GPR40-deficient mice compared with wild-type mice 2 h after zymosan injection. At this early time point the only immune cell type that can infiltrate inflamed tissue and contribute to pain are neutrophils.^32^

It has been shown before that neutrophils express GPR40 and that a GPR40 agonist can enhance neutrophil migration and function in the context of infection.^33^ Zymosan injection mimics an infection, so the mechanism identified by Souza et al., may also be relevant during zymosan-induced inflammation.

One may speculate that in GPR40-deficient mice the neutrophils are recruited later to the site of inflammation and their pronociceptive influence on peripheral sensitization and thermal hypersensitivity in early inflammatory pain is delayed in the absence of GPR40. At later time points, the number of neutrophils that have infiltrated the inflamed tissue may be high enough to mediate thermal hypersensitivity in GPR40-deficient mice. This could explain our experimental observations.

TRPV1 may have an intrinsic thermodynamic instability that causes partial unfolding of the protein in response to noxious heat as reported recently.^34^ While this could explain the discrete thermal sensing range of the channel, it may also be a protective mechanism for TRPV1-positive neurons to prevent excessive TRPV1 activity in response to heat sensations and to reduce potential subsequent damage. Peripheral sensory neurons have the exquisite task of detecting noxious stimuli and being constantly in vigilant mode, but also responding to a stimulus rapidly once it exceeds a certain threshold. Returning to the homeostatic vigilant mode after an excitation by noxious stimuli has usually been attributed to intrinsic mechanisms within neurons. Our study indicates that lateral neuron-to-neuron communication by paracrine signaling mediators may be an important additional regulatory mechanism that can reduce exacerbated neuronal response.

### Limitations of the study

In this study, we suggest that the capsaicin-mediated oleic acid release from sensory neurons acts as a protective and feedback mechanism, preventing sensory neurons from nociceptive overstimulation via the GPR40/CaN/TRPV1-axis. There are still open questions that could not be answered yet. First, it is unclear whether oleic acid is also released in chronic inflammatory or neuropathic pain and whether it can alleviate these pain states as well indicating that more *in vivo* pharmacology is necessary for a detailed understanding of this process. Second, we do not know whether oleic acid also autocrine actions has independent of its paracrine signaling that may be independent of GPR40. Third, we do not know whether sex differences have any effect on oleic acid-mediated release and desensitization. These questions should be addressed by further studies.

## STAR★Methods

### Key resources table

**Table:** 

REAGENT or RESOURCE	SOURCE	IDENTIFIER
**Antibodies**		

anti GPCR GPR40	Abcam	ab236285
anti NeuN-Cy3	Merck	#MAB377C3
anti-IP-1	Cisbio Bioassays	IP-One assay kit 62IPAPEC
anti rabbit-AF488	Abcam	ab150077

**Chemicals, peptides, and recombinant proteins**		

0.1% Triton X-100	Sigma-Aldrich	9036-19-5
paraformaldehyde	Carl Roth	200-001-8
ATP-Mg salt	Sigma-Aldrich	A9187
B-277™ Supplement	Gibco	17504001
bovine serum albumin fraction V	Carl Roth	232-936-2
bradykinin	Tocris	3002
CaCl_2_	Sigma-Aldrich	C5080-500G
bromoenol lactone	Sigma-Aldrich	B1152
capsaicin	Sigma-Aldrich	404-86-4
coelenterazine	Promega	S2001
collagenase type IV	Sigma-Aldrich	L2020
cytosolic phospholipase A2 (cPLA2) inhibitor AACOCF_3_	Tocris	1462
D-(+)-Glucose	Sigma-Aldrich	G8270-1KG
DAPI	Sigma-Aldrich	28718-90-3
dimethyl sulfoxide	Sigma-Aldrich	472301-500ML
Dispase II	Roche	04942078001
EGTA	Carl Roth	200-651-2
FFAR1 reference agonist GW9508	Cayman	#Cay10008907
Fura-2-AM	Biotum	50033
gentamycin (50 mg/mL)	Gibco	15750037
GPR40 antagonist DC260126	Tocris	5357
HEPES	PanReac AppliChem	A1069
KCl	Sigma-Aldrich	31248
L-glutamine	Sigma-Aldrich	G7513
LiCl	Carl Roth	231-212-3
Lipofectamine 2000	ThermoFisher	11668019
MgCl_2_	Sigma-Aldrich	M2670-500G
NaCl	Sigma-Aldrich	31434-5KG-R
NaH_2_PO_4_	Sigma-Aldrich	71507
NaHCO_3_	Carl Roth	205-633-8
Oleic acid	Sigma-Aldrich	O1008-1G
penicillin-streptomycin	ThermoFisher	15140122
poly-L-lysine	Sigma-Aldrich	P2636-1G
potassium gluconate	Sigma-Aldrich	P1847
sucrose	Carl Roth	200-334-9

**Deposited data**		

source data	this manuscript	N/A

**Experimental models: Cell lines**		

CHO-K1 cells	DSMZ	ACC 110

**Experimental models: Organisms/strains**		

C57BL/6NRj mice	Janvier, Charles River	N/A
GPR40 deficient mice with C57BL/6NRj background	Provided by Prof. Klaus Scholich	N/A

**Recombinant DNA**		

pSBbi-Bla	Addgene	#60526
human FFAR1 ORF Clone (BC120944)	Creative Biogene	CDCS412194
pCMV(CAT)T7-SB100	Addgene	#34879
pcDNA3.1	Invitrogen	V79520

**Software and algorithms**		

pClamp 10.3 software	Molecular Devices	N/A
Minianalysis ver. 6.0.3	Synaptosoft	N/A
ZEN 2.3 pro	Zeiss	N/A
Analyst v1.7.1	Sciex	N/A
Analyst TF v1.7.1	Sciex	N/A
MultiQuant v3.02 v3.03 software	Sciex	N/A
GraphPad PRISM version 9.0	Dotmatics	N/A

**Other**		

Neurobasal™ Medium	Gibco	21103–049
Ham′s F-12 Nutrient Mix with GlutaMAX supplement	Gibco	#31765

### Resource availability

#### Lead contact

Further information and requests for resources and reagents should be directed to and will be fulfilled by the lead contact, Marco Sisignano Goethe University Frankfurt, University Hospital, Institute of Clinical Pharmacology, Theodor-Stern-Kai 7, 60590 Frankfurt am Main, Germany, Phone: +49 (0)69-6301-87819 Marco.Sisignano@med.uni-frankfurt.

#### Materials availability

This study did not generate new unique reagents.

#### Data and code availability

• Accession numbers are listed in the key resources table. All the data published in this manuscript is available.
• This paper does not report original code.
• Any additional information required to reanalyze the data reported in this paper as well as LC-HRMS raw data is available from the lead contact upon request.

### Experimental model and study participant details

#### Mice

We used male wild-type C57BL/6NRj mice that were 7–12 weeks old that were purchased from commercial breeding companies (Janvier, Le Genest-Saint-Isle, France; Charles River, Wilminton, USA). GPR40 deficient mice were bred in-house at an age of 9–15 weeks.^35^ The animals were then bred in the laboratory animal facility of the University Hospital of Goethe-University. All experiments involving animals were approved by the local Ethics Committees for Animal Research (Darmstadt, Germany) under the permit numbers FK/1113 and FU/2018.

#### Cell lines

CHO-K1 cells were purchased from DSMZ (ACC 110) and maintained at 5% CO_2_ and 37°C in the full growth medium (Ham′s F-12 Nutrient Mix with GlutaMAX supplement; Gibco #31765) supplemented with 10% fetal bovine serum (FBS; capricon) and 100 units/mL penicillin and 100 μg/mL streptomycin (Gibco). For splitting cells were harvested using Trypsin/EDTA.

#### Primary cell cultures

For primary cell cultures, male mice wild-type C57BL/6NRj and GPR40 deficient mice with C57BL/6NRj background were used for cell isolation. The mice were 7–12 weeks or 9–15 weeks old, respectively. The wild type animals were purchased from commercial breeding companies (Janvier, Le Genest-Saint-Isle, France; Charles River, Wilminton, USA) and GPR40 deficient mice were bred in-house and were kindly provided by Prof. Klaus Scholich.^35^

### Method details

#### Ethics statement

The procedures were performed in accordance with the ethical standards of the Guide for the Care and Use of Laboratory Animals of the National Institutes of Health and the ARRIVE 2.0 guidelines.^36^ Furthermore, all animal experiments were performed according to the recommendations of the Preclinical Pain Research Consortium for Investigating Safety and Efficacy (PPRECISE) Working Group.^37^ We made all efforts to avoid and minimize animal suffering where it was possible. The experimenter was blinded during all behavioral experiments.

#### Culturing primary DRG neurons

Dorsal root ganglia (DRG) were isolated from mice as described previously.^38^ Murine DRGs from thoracic to lumbar segments were dissected and transferred to cooled Hanks’ Balanced Salt Solution (HBSS) with CaCl_2_ and MgCl_2_ (Gibco). Then the DRGs were incubated with Neurobasal Medium (Invitrogen) containing 500 U/mL collagenase (Sigma-Aldrich) and 2.5 U/mL dispase II (Roche) for 75 min at 37°C. The collagenase/dispase solution was then removed and the cells were washed twice with Neurobasal Medium containing 10% inactivated fetal bovine serum (FBS), 1% penicillin/streptomycin (Gibco). Then the cells were incubated with 0.05% Trypsin (Gibco) for 10 min and again washed twice. The cells were then resuspended with Neurobasal Medium containing L-glutamine (2 nM; Sigma-Aldrich), penicillin (100 U/mL; Gibco), streptomycin (100 μg/mL; Gibco), B-27 (Gibco) and gentamycin (50 μg/mL; Gibco). Afterward the cells were mechanically dissociated by gentle aspiration, plated on poly-L-lysine (Sigma-Aldrich) coated glass coverslips and incubated for 2 h at 37°C to allow the cells to adhere to the coverslip surface. Finally, 2 mL of Neurobasal Medium with L-glutamine, penicillin/streptomycin, B-27 and gentamicin were added, and the neurons were incubated over night at 37°C and 5% CO_2_.

#### Calcium imaging

DRG neurons were stained with Fura-2-AM (Biotium) for 45–90 min at 37°C. For experiments in which the GPR40 antagonist DC260126 (Tocris) was used, the cells were preincubated together with the Fura-2-AM staining solution. From this point on a freshly prepared Ringer’s solution (145 mM NaCl, 1.25 CaCl_2_, 1 mM MgCl_2_, 5 mM KCl, 10 mM D-glucose, 10 mM HEPES; pH 7,3) was used for all washing and preparation of perfusion solutions. Sensitization measurements were performed by perfusing twice with capsaicin (100 nM, 12–17 s; Sigma-Aldrich). Before the second capsaicin perfusion, the cells were perfused with the substance of interest, such as bradykinin (100 nM, 4 min; Tocris), oleic acid (0,2–10 μM, 2–4 min; Sigma-Aldrich), inhibitors, agonists or any combination of the mentioned substances. To confirm that the recorded signals originated from neurons, additionally, the cells were stimulated with 50 mM KCl.

#### Immunohistochemistry

After Calcium Imaging measurements, each coverslip with DRG neurons was immediately placed into a 24-well plate und fixed with 2% paraformaldehyde (PFA; Roth) in PBS (Gibco) for 30 min. Then PFA was removed and PBS added. The next day the cells were washed three times in the 24-well pate, permeabilized with PBST (PBS and 0.1% Triton X-100; Sigma-Aldrich) for 25 min and blocked with 3% BSA in PBST for 45 min at room temperature. Then the cells were stained with primary antibodies anti-NeuN-Cy3 (Merck, #MAB377C3, mouse, 1:400) and anti-GPCR GPR40 (Abcam, ab236285, rabbit, 1:200) over night at 4°C. Afterward, the cells were washed three times with PBST and the secondary antibody anti-rb-AF488 (Abcam, ab150077, goat, 1:1000) was applied for 2 h at room temperature. After washing three times and staining with DAPI (1 μg/mL; Sigma Aldrich) for 10 min at room temperature, the cells were washed again three times with PBST and once with PBS. The cover slips where then placed on microscope slides and fixed with DPX Mountant. Finally, the slides were analyzed by an inverted Zeiss Observer.Z1 Microscope using the Software ZEN 2.3 pro.

#### Cell transfection and determination of [Ca^2+^]_i_

CHO cells were seeded in 96 well plates with white walls and transparent bottom at a density of 30000 cells/well. Twenty-four hours later, they were transfected with plasmids containing cDNA encoding a calcium-sensitive bioluminescent fusion protein consisting of aequorin and GFP^39^ and the human FFAR1 or just the carrier vector, pcDNA3.1 (Invitrogen) by using Lipofectamine 2000 (ThermoFisher) following manufacturer’s indications. Forty-eight hour later, cells were retrieved form the incubator, and growth medium was replaced with 5 μM coelenterazine (Promega) in Hank’s balanced salt solution (HBSS) containing 2 mM calcium and 10 mM glucose for 2 h at 37°C in a CO2-free incubator. Measurements were performed by using a luminometric plate reader (Flexstation 3, Molecular Devices). The area under each calcium transient was calculated by using SoftMaxPro 6 software and expressed as area under the curve (AUC).

#### Generation of stable CHO-K1 cell line constitutively overexpressing *h.s.* FFAR1

A stable cell line constitutively overexpressing FFAR1 was generated using the sleeping beauty method.^40^ The natural CDS (coding DNA sequence) for *h.s.* FFAR1 (uniprot O14842) was cloned into pSBbi-Bla (addgene #60526) using BC120944.1 as template, which encodes the natural variant with Arg211 changed to His. Stable insertion of the expression cassette into the genome of CHO-K1 cells (DSMZ, ACC 110) was then facilitated using the sleeping beauty method with the transposase expressed from the cotransfected plasmid pCMV(CAT)T7-SB100 (addgene #34879), and cells were selected with Blasticidin (resistance procured by pSBbi-Bla). Expression of FFAR1 is governed by the constitutively active EF1a promoter.

#### IP-one assay

Ligand dependent activation of FFAR1 was probed using cell-based assays in which the accumulation of IP-1 (inositol monophosphate) as a downstream effect was detected in a displacement assay based on HTRF (homogeneous time-resolved FRET). For detection of IP-1 the utilized FRET pair was composed of FRET acceptor coupled IP-1 and Terbium cryptate coupled anti-IP-1 antibody (62IPAPEC, IP-One assay kit, Cisbio Bioassays). One day ahead of performing the assay, CHO-K1 cells stably overexpressing FFAR1 were seeded into white tissue culture 384-well plates (Thermo Fisher #164610) in 50 μL full growth medium at 12.500 cells/well. After overnight incubation at 37°C and 5% CO_2_, the medium was removed and cells were washed four times with IP-One stimulation buffer (146 mM NaCl, 4.2 mM KCl, 1 mM CaCl_2_, 0.5 mM MgCl_2_, 50 mM LiCl, 5.5 mM D-glucose, 0.1% (w/v) fatty acid-free bovine serum albumin fraction V (Carl Roth) buffered with 10 mM HEPES at pH 7.4 (NaOH)) using a Tecan HydroSpeed plate washer (Tecan Deutschland GmbH). Thereafter, the compounds at the indicated concentrations and a total of 0.5% DMSO were added to the cells, and the plate was sealed and incubated at 37°C for the stimulation period. After 90 min, the cells were lysed by addition of the detection agents prepared in lysis buffer according to the manufacturer′s instructions. The concentration of IP-1 produced by the cells was calculated from a standard curve using dilutions of unlabeled IP-1 in buffer without cells. A full dose-response curve with FFAR1 reference agonist GW9508 (cayman #Cay10008907) served as control for assay performance. Experiments were performed in parallel on wild-type CHO-K1 cells in order to exclude general cellular effects as well as assay artifacts. Cells overexpressing FFAR1 showed a strong response after treatment with GW9508, while wild-type CHO-K1 cells did not show any response.

#### Whole-cell patch clamp recordings in spinal cord slices

C57BL/6 mice (aged 5 weeks of both sexes) were anesthetized with urethane (1.5–2.0 g/kg, i.p.). After a dorsal laminectomy, the lumbo-sacral segment of the spinal cord was removed and placed into the pre-oxygenated, ice-cold cutting solution (in mM: sucrose 240, NaHCO_3_ 25, KCl 2.5, NaH_2_PO_4_ 1.25, CaCl_2_ 0.5, MgCl_2_ 3.5). The mice were then immediately euthanized by decapitation. After removal the arachnoid membrane, the spinal cord was placed in an agar block and mounted on a metal stage. A transverse slice (400 μm thick) was cut on a vibrating microslicer (VT1200S, Leica). The slices were incubated for 30 min in artificial cerebrospinal fluid (ACSF) equilibrated with 95% O_2_ and 5% CO_2_ gas mixture. The ACSF contained (in mM): NaCl 126, KCl 3, CaCl_2_ 2.5, MgCl_2_ 1.3, NaH_2_PO_4_ 1.25, NaHCO_3_ 26, and D-glucose 11.

The spinal cord slice was placed in a recording chamber and perfused at a flow rate 2–4 mL/min with ASCF equilibrated with 95% O_2_ and 5% CO_2_ gas mixture. Whole-cell patch-clamp recordings were made from substantia gelatinosa (SG) neurons with patch pipette electrodes having a resistance of 5–8 MΩ, as previously reported.^41^ We focused on outer lamina IIo neurons, as these neurons are predominately excitatory (expressing somatostatin) and form a neurocircuit with capsaicin-sensitive C-fiber afferent and lamina I project neurons.^42^^,^^43^^,^^44^^,^^45^^,^^46^ All experiments were performed in voltage-clamp mode. The holding potential was set to −70 mV when recording of EPSCs. The patch pipette solution contained (in mM): potassium gluconate 135, CaCl_2_ 0.5, MgCl_2_ 2, KCl 5, EGTA 5, HEPES 5, ATP-Mg salt 5. Drugs were dissolved in ASCF.

Signals were amplified using an Axopatch 700B amplifier (Molecular Devices) and were filtered at 2 kHz and digitized at 5 kHz. Data were collected and analyzed using pClamp 10.3 software (Molecular Devices). Spontaneous EPSCs (sEPSCs) were analyzed using Minianalysis ver. 6.0.3 (Synaptosoft).

#### Measurement of lipid release from DRG cultures

DRG neurons isolated from four wild-type mice were cultured overnight in a 48-well plate coated with poly-L-lysine. The cells were washed with HBSS and then preincubated with either with the calcium-independent phospholipase A2 (iPLA2) inhibitor bromoenol lactone (BEL, 1 μM; Sigma-Aldrich) or the cytosolic phospholipase A2 (cPLA2) inhibitor AACOCF3 (20 μM; Tocris) for 1 h and then stimulated with capsaicin (500 nM). The supernatant was then taken for analysis. The cells were treated with accutase (Sigma-Aldrich) for 5 min and then scraped to detach and lysate the cells. 100 μL of HBSS were then added to stop the accutase activity. Both, the supernatant and the lysates were stored at −80°C until further analyses.

#### Acute inflammatory pain model

WT and GPR40-KO mice were in a model of acute inflammatory pain for behavioral pain testing. Thereby peripheral inflammation was induced by injecting 20 μL of 3 mg/mL zymosan in PBS (Gibco) into the plantar side of one hind paw. Mice were additionally treated with oleic acid (50 μM) or vehicle (DMSO 1% (v/v)).

#### Dynamic plantar test for mechanical threshold determination

For the assessment of the mechanical pain thresholds, the dynamic plantar test as described before.^47^ Briefly, a steel rod pushed into the midplantar area of the hind paw. The force with which the steel rod was pushing was ascending in a linear way (0–5 g over 10 s, in 0.5 g/s intervals) and the threshold was set to 5 g and 20 s. The withdrawal threshold was then presented as paw withdrawal latency (PWL) to the mechanical stimulus in seconds.

#### Radiant heat test for thermal threshold determination

Thermal pain thresholds were measured using a Hargreaves apparatus as described before.^47^ Briefly, a heat stimulus was introduced to the midplantar region of the paw using a radiant heat device, consisting of a plastic cage in which the mouse was placed and a high-intensity projector lamp. During mechanical and thermal allodynia, a fast withdrawal of the paw was considered a nociceptive response. Thereby the mouse had to show cognitive awareness of pain for the reaction to qualify as a valid response.

Baseline testing was performed a day before the zymosan injection. Then mechanical and thermal testing was performed on the day of zymosan injection one to 6 h after injection and afterward every day for three consecutive days. On the third day, mice were then sacrificed to harvest lumbar DRGs and spinal cord tissue as well as sciatic nerves.

#### Lipid measurement by LC-HRMS

The lipid analysis via LC-QTOFMS was performed as previously described.^48^ To 140 μL of cell supernatant, 150 μL of internal standards in methanol and 500 μL of MTBE were added and vigorously mixed. After centrifugation for 5 min at 20,000 g at ambient temperature, the upper phase was transferred and the lower phase reextracted with 200 μL of MTBE:methanol:water (10:3:2.5 v/v/v, upper phase) followed by vortexing and centrifugation (5 min at 20,000 g). The combined organic layers were split into two aliquots for measurement in positive and negative ionization mode respectively and dried under a nitrogen stream at 45°C. The dried samples were stored at −80°C and redissolved in 40 μL of methanol before analysis.

LC-MS analysis was conducted on Nexera X2 system (Shimadzu Corporation) coupled to a TripleTOF 6600 with a DuoSpray ion source using electrospray ionization (both Sciex). A Zorbax RRHD Eclipse Plus C8 1.8 μm 50 × 2.1 mm ID column (Agilent) with a precolumn of the same material was used for peak separation with 10 mM ammonium formate and 0.1% formic acid in water as mobile phase A and 0.1% formic acid in acetonitrile: isopropanol (2:3, v/v) as mobile phase B. For measurement in negative ionization mode mobile phase A was switched to 1 mM ammonium formate and 0.1% formic acid in water. The MS1 Scan covered a mass range from 100 to 1000 m/z and the six data dependent MS/MS-spectra per cycle covered a mass range from 50 to 1000 m/z with a collision energy of 40 ± 20V. Sample acquisition was performed with Analyst TF v1.7.1 and the data further processed using MultiQuant v3.02 software (both Sciex). The peak areas were normalized using the average signal of the internal standards (Table S1).

#### Determination of fatty acids by LC-MS/MS

Fatty acids were analyzed using liquid chromatography tandem-mass spectroscopy (LC-MS/MS). The LC-MS/MS SYSTEM for analysis of fatty acids consisted of a 5500 QTrap mass spectrometer (Sciex), operating in negative electrospray ionization mode, an Agilent 1260 HPLC system (Agilent) and an HTC Pal autosampler (Chromtech).

Tissue samples were homogenized using a Mixer Mill MM400 (Retsch) with 5 zirconium dioxide balls in 200 μL ethanol:water (1:3, v/v) at 30 Hz for 2 × 4 min. The analysis and extraction were performed by liquid-liquid extraction: 5 μL of tissue homogenate were used for the determination of arachidonic acid, oleic acid and linoleic acid (+100 μL HBSS Medium). Samples were gently mixed with 20 μL of methanol containing 0.1% BHT, 20 μL of internal standard solution, 100 μL EDTA 0.15 mM solution in water and extracted twice with 600 μL ethyl acetate. Samples for calibration curve and quality control were prepared similarly: 150 μL HBSS Medium, 20 μL of standard solution, 20 μL internal standard solution and 100 μL EDTA 0.15 mM solution in water were mixed and extracted with ethyl acetate.

The organic phase was removed at 45°C under a gentle stream of nitrogen. The residues were reconstituted in 50 μL 60:40 v/v 10 mM ammonium acetate:(acetonitrile/isopropyl alcohol/10 mM ammonium acetate (55:40:5, v/v/v)) prior to injection into the LC-MS/MS system.

Chromatographic separation was achieved using a Zorbax Eclipse Plus C8 RRHD 1.8 μm 50 × 2.1 mm ID column (Agilent) and a 11.5 min linear gradient with a flow rate of 0.5 mL/min. The mobile phases were 10 mM ammonium acetate in water and acetonitrile/isopropyl alcohol/10 mM ammonium acetate (55:40:5, v/v/v).

All data was acquired using Analyst software v1.7.1 and quantification was performed by MultiQuant software v3.03 (both Sciex) using the internal standard method (isotope-dilution mass spectrometry). Calibration curves were calculated by linear regression with 1/x weighting.

### Quantification and statistical analysis

All data are presented as mean ± SEM. To determine statistically significant differences between two groups Student’s t test was carried out and for comparing more than two groups one-way ANOVA was used. Comparisons with more than two groups and different conditions were performed with two-way ANOVA following Tukey’s multiple comparisons test. For analyzing behavioral data, we used two-way ANOVA following Bonferroni’s multiple comparisons. A *p*-value of *p* < 0.05 was considered as statistically significant.
