# Supplementary material for: Oleic acid released by sensory neurons inhibits TRPV1-mediated thermal hypersensitivity via GPR40
Source: iScience. 2024 Jul 20;27(8):110552. doi: 10.1016/j.isci.2024.110552 (PMC11338150; doi:10.1016/j.isci.2024.110552)
Supplement: Document S1. Figures S1–S3 and Table S1 [file mmc1.pdf]

iScience, Volume *27*

Supplemental information

Oleic acid released by sensory neurons inhibits TRPV1-mediated thermal hypersensitivity via GPR40

Maksim Sendetski, Saskia Wedel, Kenta Furutani, Lisa Hahnefeld, Carlo Angioni, Jan Heering, Béla Zimmer, Sandra Pierre, Alexandra-Maria Banica, Klaus Scholich, Sorin Tunaru, Gerd Geisslinger, Ru-Rong Ji, and Marco Sisignano

SUPPLEMENTAL DATA

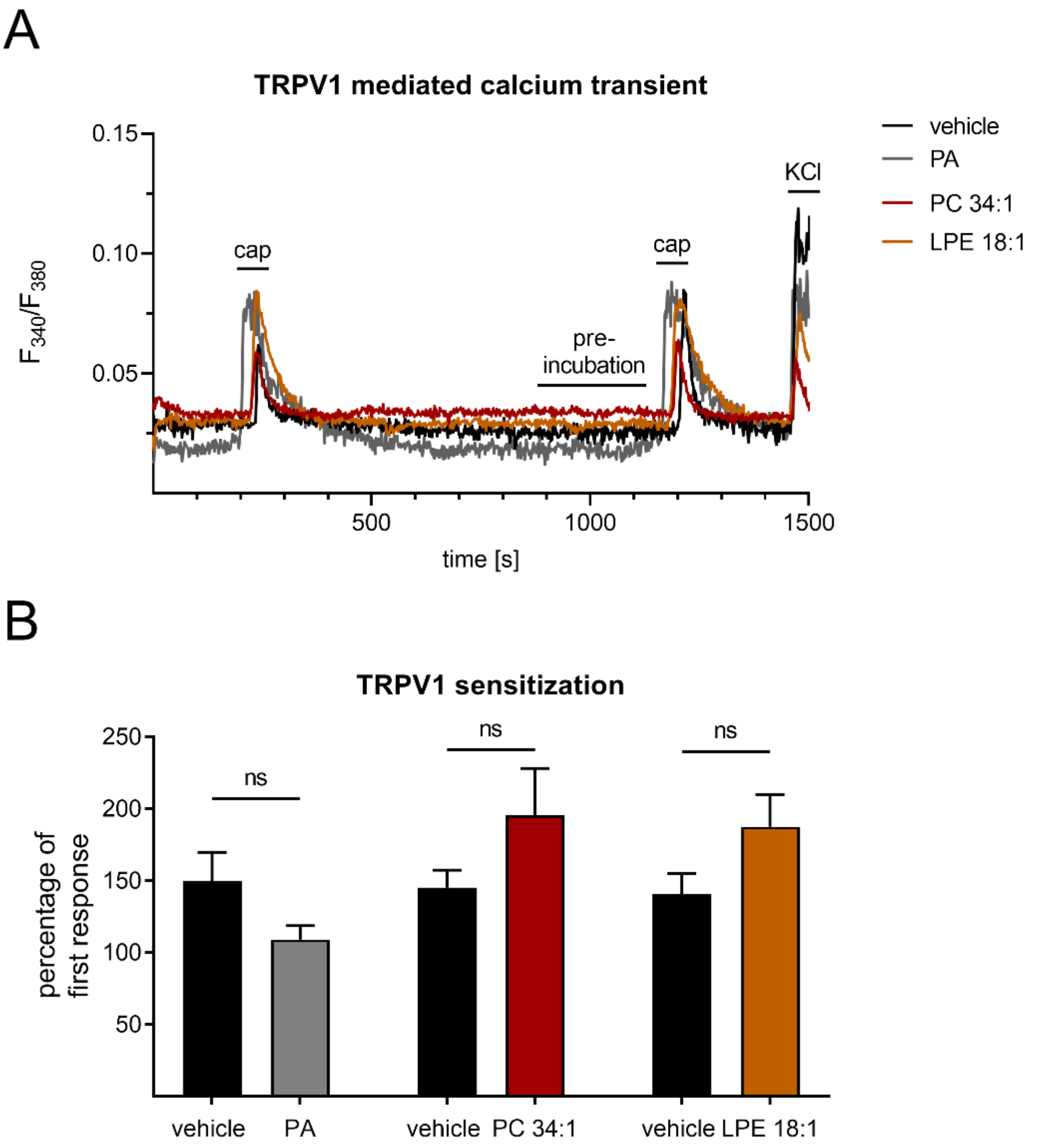

Figure S1: Influence of palmitoleic acid, PC 34:1 and LPE 18:1 on TRPV1 mediated calcium transients in primary sensory neurons related to Figure 1. (A) Representative traces of TRPV1 mediated calcium transients after preincubation with palmitoleic acid (1 µM), PC 34:1 (1 µM) and LPE 18:1 (1 µM) for two minutes. **(B)** Quantification of TRPV1 mediated calcium transients after preincubation with 1 µM of the respective lipids. The data represent the mean ± SEM of 22-75 sensory neurons. *$p<0.05$ two-way ANOVA with Sidak´s multiple comparisons test. Abbreviations: cap: capsaicin, PA: palmitoleic acid, PC: phosphocholine, LPE: lysophosphatidyl-ethanolamine

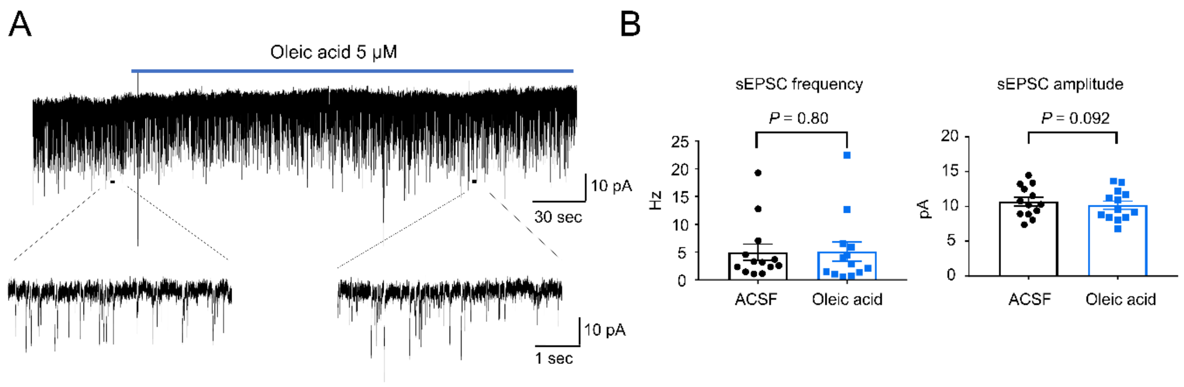

Figure S2: Effect of oleic acid on sEPSCs in comparison to ACSF in neurons related to Figure 1. (A) Representative traces of sEPSCs with and without the perfusion of oleic acid (5 µM). **(B)** Quantification of oleic acid- induced fold change of sEPSC frequency (left) and amplitude (right). The data represent the mean of 18-19 neurons from 3 mice ± SEM. *p<0.05 unpaired t-test. Abbreviations: ACSF: artificial cerebrospinal fluid, sEPSC: spontaneous excitatory postsynaptic current

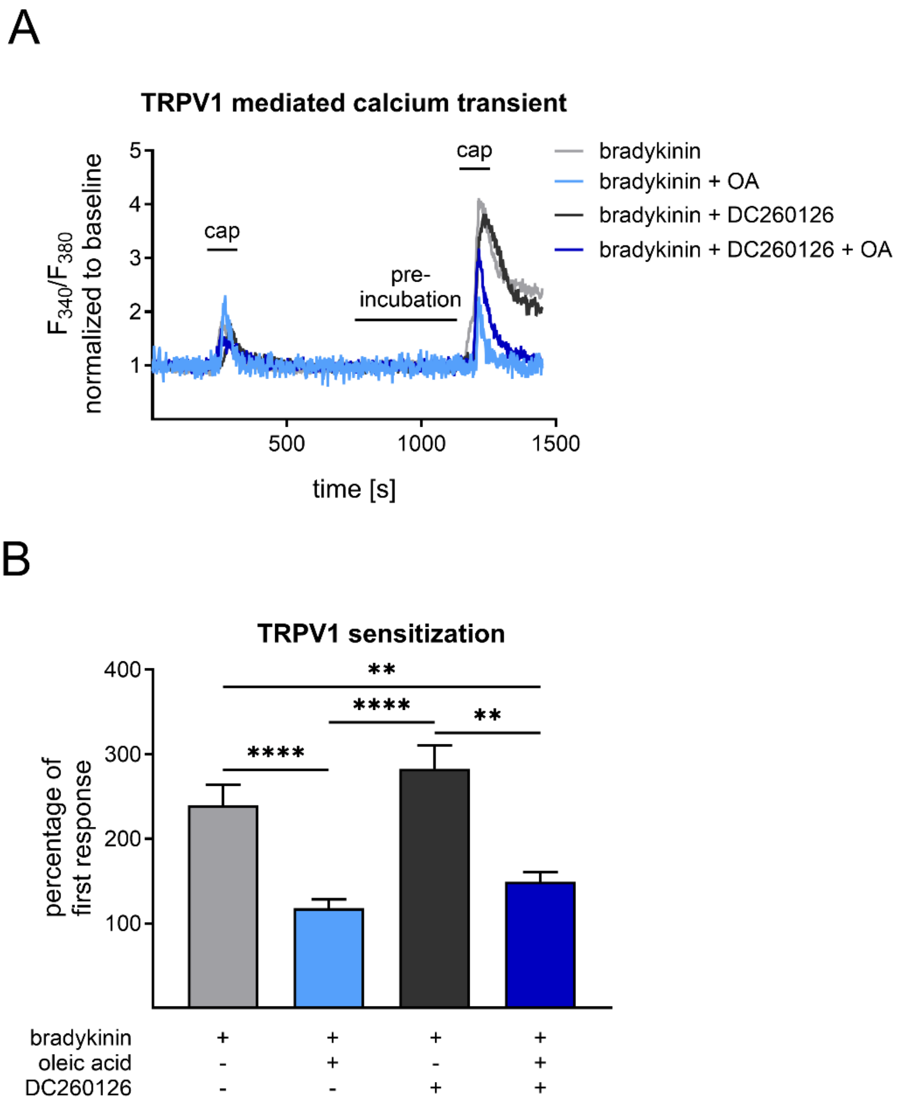

Figure S3: Influence of the GPR40 antagonist DC260126 on TRPV1 sensitization in primary sensory neurons related to Figure 3. (A) Representative traces of TRPV1 mediated calcium transients after preincubation with bradykinin (100 nM) together with or without oleic acid (1 µM) for two minutes. Before the measurement, the sensory neurons were incubated with or without the GPR40 antagonist DC260126 (20 µM) for one hour. **(B)** Quantification of TRPV1 mediated calcium transients. The data represent the mean ± SEM of 23-75 sensory neurons. *$p<0.05$ unpaired t-test with Welch´s correction. Abbreviations: cap: capsaicin, OA: oleic acid

Table S1: Internal standard working solution for LC-HRMS analysis related to the STAR Methods

internal standard	concentration (ng/ml)
Ceramide C16-d7	1.67
Ceramide C17	1.67
Ceramide C18-d3	1.67
Ceramide C24:1-d7	1.67
Ceramide C24-d4	1.67
Ceramide d18:0/18:0-d3	1.67
Glucosylceramide C18-d5	1.67
Lactosylceramide C16-d3	1.67
Lactosylceramide C17	3.33
Sphinganine-d7	10.00
Sphingosine-d7	10.00
LPC 13:0	13.33
Sphingosine-1-phosphate-d7	13.33
Stearic acid-d35	66.67
